# Supplementary material for: Identification of Freezing-Responsive microRNAs and Their Targets in Chinese Jujube by Small RNA and Degradome Sequencing
Source: Int J Mol Sci. 2026 Sep 4;27(17):7912. doi: 10.3390/ijms27177912 (PMC13566890; doi:10.3390/ijms27177912)
Supplement: Supplementary file 1 [file ijms-27-07912-s001.zip › Supplementary Material S2/novel_miR_208.pdf]

Provisional ID : novel_miR_208
Score total : 0.7
Score for star read(s) : -1.3
Score for read counts : 0
Score for mfe : 0.4
Score for randfold : 1.6
Score for cons. seed :
Total read count : 5010
Mature read count : 2845
Loop read count : 28
Star read count : 2137

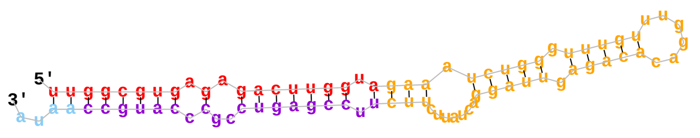

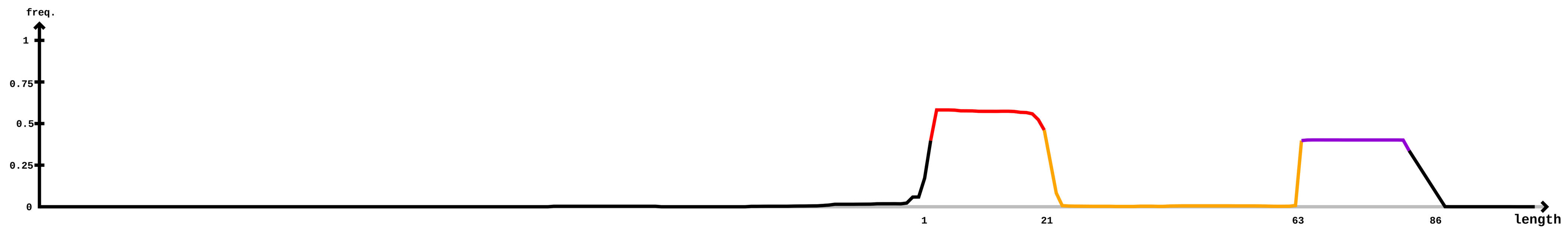

Mature Star

```
5'- agcgucacgugauaacgcguccuuugcuuuuuauuuuuuauuugaaauucaaaaaaauaauaauaaagauauauuuauauaauuguggaugugaaagaagaacuacuaccacuacagagagauuuuuuuaugaggcugagaaaauuguuggcgugagagacuugguagaaaucuggguuuguuuggacacagaguuaggacuauucuucuuccgaguccgcccaugccaauaauuuccuccgucuuuuuc -3' obs
    agcgucacgugauaacgcguccuuugcuuuuuauuuuuuauuugaaauucaaaaaaauaauaauaaagauauauuuauauaauuguggaugugaaagaagaacuacuaccacuacagagagauuuuuuuaugaggcugagaaaauuguuggcgugagagacuugguagaaaucuggguuuguuuggacacagaguuaggacuauucuucuuccgaguccgcccaugccaauaauuuccuccgucuuuuuc     exp
    .((((........))))(((((((((((((((.(((.(((((...))))).))).))))).)))).((((((((((....))))))))))..................))))).)))........(((((.(((.((((((((((((((.(.(((((((.((((.(((((.(((((......))))).))))).......)))).)))))))..).))))))))))))))).))).)))))....
```

	reads	mm	sample
guggaugugaaagaagaa	6	0	JT-2
guggaugugaaagaagaa	9	0	JT-3
agagauuuuuuuaugaggcugagaa	5	0	JT-2
agagauuuuuuuaugaggcugagaa	8	0	JT-3
agauuuuuuuaugaggcugagaaa	2	0	LT-3
gauuuuuuuaugaggcugagaa	1	0	LT-1
uuuuuaugaggcugagaaaau	1	0	JCK-1
uuuuuaugaggcugagaaaau	1	0	JCK-2
uuuuuaugaggcugagaaaauug	3	0	JCK-2
uuuuaugaggcugagaaaauuguuggcgug	2	0	LT-1
uuuaugaggcugagaaaauuguuggcgug	1	0	JT-1
uuaugaggcugagaaaauug	1	0	LT-1
uuaugaggcugagaaaauuguuggcgu	1	0	LT-1
uuaugaggcugagaaaauuguuggcgu	1	0	LT-2
uaugaggcugagaaaauuguuggcgug	2	0	LT-1
augaggcugagaaaauuguuggc	2	0	LT-3
augaggcugagaaaauuguuggc	1	0	LT-1
augaggcugagaaaauuguuggU	1	1	LT-2
augaggcugagaaaauuguuggcg	5	0	JT-2
augaggcugagaaaauuguuggcg	3	0	JT-3
ugaggcugagaaaauuguugg	3	0	LCK-3
ugaggcugagaaaauuAuugg	1	1	JCK-3
ugaggcugagaaaauuguugg	6	0	LT-2
ugaggcugagaaaauuguuggc	1	0	JCK-3
ugaggcugagaaaauuguuggc	1	0	JCK-2
ugaggcugagaaaauuguuggcgug	1	0	JCK-1
ugaggcugagaaaauuguuggcgugaga	1	0	LT-1
gaggcugagaaaauuguuggc	17	0	LCK-3
gaggcugagaaaauuguuggc	1	0	LT-2
gaggcugagaaaauuguuggcg	2	0	LT-3
gaggcugagaaaauuAuuggcgug	1	1	JCK-2
gaggcugagaaaauuAuuggcgug	2	1	JCK-3
gaggcugagaaaauuguuggcgug	2	0	LT-3

Mature Star

agcgucacgugauaacgcguccuuugcuuuuuauuuuuuauuugaaauucaaaaaaauaauaauaaagauauauuuauauaauuguggaugugaaagaagaacuacuaccacuacagagagauuuuuuuaugaggcugagaaaauuguuggcgugagagacuugguagaaaucuggguuuguuuggacacagaguuaggacuauucuucuuccgaguccgcccaugccaauaauuuccuccgucuuuuc

gaggcugagaaaauuguuggcgug	1	0	JCK-1
cugagaaaauuguuggcgug	1	0	LT-3
cugagaaaauuguuggcguga	1	0	LT-3
ugagaaaauuguuggcgugagagacu	1	0	LT-3
gagaaaauugAuggcgugagaga	1	1	LT-3
agaaaauuguuggcgugagag	3	0	LT-3
agaaaauuguuggcgugagag	2	0	LT-1
agaaaauuguuggcgugagagacu	6	0	LCK-3
gaaaauuAuuggcgugagagacuuggua	1	1	JCK-1
aaauuAuuggcgugagagacuugg	1	1	JCK-1
aauuguuggcgugagagac	6	0	LCK-3
aauuguuggcgugagagac	1	0	LT-1
aCuuguuggcgugagagacu	1	1	LT-3
aauuguuggcgugagagacuugg	2	0	JCK-3
aauuguuggcgugagagacuugg	1	0	JCK-2
auuguuggcgugagagac	2	0	LT-2
auuAuuggcgugagagacu	1	1	JCK-2
auuguuggcgugagagacuug	1	0	LT-3
auuguuggcgugagagacuug	2	0	LT-1
auuAuuggcgugagagacuuggua	1	1	JCK-1
auuAuuggcgugagagacuuggua	2	1	JCK-2
auuguuggcgugagagacuuggua	4	0	LT-2
auuguuggcgugagagacuuggua	3	0	LT-1
auuguuggcgugagagacuuggua	4	0	LCK-3
auuguuggcgugagagacuuggua	1	0	JCK-1
auuAuuggcgugagagacuuggua	2	1	JCK-3
auuguuggcgugagagacuuggua	4	0	LT-3
uuguuggcgugagagacu	3	0	LT-3
uuguuggcgugagagacu	2	0	JCK-2
uuguuggcgugagagacu	5	0	LCK-3
uuguuggcgugagagacu	2	0	LT-1
uuAuuggcgugagagacu	3	1	JCK-2
uuguuggcAugagagacu	1	1	LCK-3
uuAuuggcgugagagacu	1	1	JCK-3
uuAuuggcgugagagacuu	3	1	JCK-3
uuAuuggcgugagagacuu	1	1	JCK-2
uuguuggcgugagagacuu	2	0	LT-1
uuguuggcgugagagacuu	1	0	JCK-1
uuguuggcgugagagacuug	8	0	LCK-3
uuAuuggcgugagagacuug	1	1	JCK-2
uuguuggcgugagagacuug	2	0	LT-2
uuAuuggcgugagagacuugg	29	1	JCK-3
uuAuuggcgugagagacuugg	41	1	JCK-2
uuguuggcgugagagacuugg	7	0	LCK-3
uuAuuggcgugagagacuugg	11	1	JT-1
uuCuuggcgugagagacuugg	1	1	JCK-2
uuguuggcgugagagacuugg	10	0	LT-2
uuguuggcgugagagacuugg	6	0	LT-3
uuguuggcgugagagacuugg	5	0	LT-1
uuAuuggcgugagagacuugg	32	1	JCK-1
uuguuggcgugagagacuugg	1	0	JCK-3
uuAuuggcgugagagacuuggu	1	1	JCK-1
uuguuggcgugagagacuuggu	1	0	JCK-2
uuguuggcgugagagacuuggu	1	0	LT-1
uuguuggcgugagagacuuggu	1	0	JCK-1
uuguuggcgugagagacuuggu	1	0	LT-2
uuguuggcgugagagacuuggu	1	0	JCK-3
uuAuuggcgugagagacuuggua	1	1	JCK-1
uuAuuggcgugagagacuuggua	3	1	JCK-2
uuguuggcgugagagacuuggua	1	0	LT-1
uuguuggcgugagagacuuggua	2	0	LT-3
uuAuuggcgugagagacuuggua	4	1	JCK-3
uguuggcgugagagacuu	1	0	LT-1
uguuggcgugagagacuugg	1	0	LT-1
uguuggcgugagagacuuggua	2	0	LT-3
Auuggcgugagagacuug	12	1	JCK-2
Auuggcgugagagacuug	7	1	JCK-3
Auuggcgugagagacuug	2	1	JT-1
Auuggcgugagagacuug	6	1	JCK-1
Auuggcgugagagacuugg	5	1	JT-3

Mature — Star

agcgucacgugauaacgcguccuuugcuuuuuauuuuuuauuugaaauucaaaaaaauaauaauaaagauauauuuauauaauuguggaugugaaagaagaacuacuaccacuacagagagauuuuuuuaugaggcugagaaaauuguuggcgugagagacuugguagaaaucuggguuuguuuggacacagaguuaggacuauucuucuuccgaguccgcccaugccaauaauuuccuccgucuuuuc

Auuggcgugagagacuugg	3	1	JCK-1
Auuggcgugagagacuugg	3	1	JCK-3
Auuggcgugagagacuugg	7	1	JT-2
Auuggcgugagagacuugg	4	1	JCK-2
Cuuggcgugagagacuuggu	1	1	JCK-2
Auuggcgugagagacuuggu	14	1	JCK-1
Auuggcgugagagacuuggu	3	1	JT-2
Auuggcgugagagacuuggu	55	1	JCK-2
Auuggcgugagagacuuggu	24	1	JCK-3
Auuggcgugagagacuuggu	3	1	JT-3
Auuggcgugagagacuuggua	81	1	JCK-1
guuggcgugagagacuuggua	2	0	LT-1
Auuggcgugagagacuuggua	171	1	JCK-2
Auuggcgugagagacuuggua	144	1	JCK-3
Cuuggcgugagagacuuggua	2	1	JCK-2
Cuuggcgugagagacuuggua	2	1	JCK-3
Auuggcgugagagacuuggua	9	1	JT-1
Auuggcgugagagacuugguag	17	1	JCK-2
Auuggcgugagagacuugguag	18	1	JCK-3
Auuggcgugagagacuugguag	20	1	JCK-1
guuggcgugagagacuugguagaa	1	0	LT-3
uuggcgugagagacuugg	4	0	JCK-3
uuggcgugagagacuugg	6	0	JCK-2
uuggcgugagagacuugg	2	0	LCK-3
uuggcgugagagacuugg	3	0	LT-3
uuggcgugagagacuugg	4	0	LT-2
uuggcgugagagacuugg	2	0	LT-1
uuggcgugagagacuuggu	24	0	LCK-2
uuggcgugagagacuuggu	18	0	JCK-1
uuggcgugagagacuuggu	15	0	LT-1
uuggcgugagagacuuggu	2	0	JT-2
uuggcgugagagacuuggu	41	0	JCK-2
uuggcgugagagacuuggu	1	0	JT-3
uuggcgugagagaAuuggu	1	1	JT-2
uuggcgugagagacuuggu	7	0	LT-3
uuggcgugagagacuuggu	11	0	JT-1
uuggcgugagagacuuggu	23	0	JCK-3
uNggcgugagagacuuggu	1	1	JCK-2
uuggcgugagagacuugUu	1	1	JCK-3
uuggcgugagagacuuggu	3	0	LCK-3
uuggcgugagagacuuggu	17	0	LT-2
uuggcgugagagacuuggu	20	0	LCK-1
uuggcgugagagacuuggua	21	0	LT-2
uuggcgugagaAacuuggua	1	1	JCK-2
uuggcgugagagacuuggua	46	0	LT-3
uuggcgugagCgacuuggua	1	1	JCK-2
uuggcgugagagacuuggua	154	0	JCK-1
uuggcgugagagacuuggua	7	0	JT-2
uuggcgugagagacuuggua	13	0	LT-1
uuggcgugagagacuugguC	2	1	LT-3
uuggcgugagagacuuggua	208	0	JCK-2
uuggcgugagagacuuggua	6	0	JT-3
uuggcgugagagacuugguC	1	1	JCK-2
uuggcgugagagacuugguU	10	1	JCK-3
uuggcgugagaUacuuggua	1	1	LT-2
uuggcgugagagacuugguU	4	1	JCK-2
uuggcgugagagacuugguU	2	1	LT-3
uuggcgugagagacCuggua	1	1	JCK-3
uuggcgugagagacuuAgua	1	1	JCK-2
uuggcgugagagacuuggua	32	0	JT-1
uuggcgugagagacuugguU	3	1	JCK-1
uuggcgugagagacuugguU	4	1	LCK-3
uuggcgugagagaAuuggua	2	1	JCK-1
uuggcgugagCgacuuggua	2	1	JT-1
uuggcgugagagacuuggua	41	0	LCK-3
uuggcgugagagacuuggua	183	0	JCK-3
uuggcgugagagacuugCua	1	1	JCK-1
uuggcgugagagaAuuggua	1	1	JCK-3
uuggcgugCgagacuuggua	1	1	JT-1
uuggcgugagagacuugguC	1	1	LT-1

Mature — Star

agcgucacgugauaacgcguccuuugcuuuuuauuuuuuauuugaaauucaaaaaaauaauaauaaagauauauuuauauaauuguggaugugaaagaagaacuacuaccacuacagagagauuuuuuuaugaggcugagaaaauuguuggcgugagagacuugguagaaaucuggguuuguuuggacacagaguuaggacuauucuucuuccgaguccgcccaugccaauaauuuccuccgucuuuuc

uuggcgugagagacuugguag	40	0	JT-1
uuggcgugagagacuugguag	27	0	JCK-3
uuggcgugagagacuugguag	13	0	LT-2
uuggcgugagagacuugguaA	3	1	JCK-3
uuggcgugagagacuugguag	7	0	LT-3
uuggcgugagagacuugguag	8	0	LT-1
uuggcgugagagacuugguag	2	0	LCK-3
uuggcgugagagacuugguaA	1	1	JCK-2
uuggcgugagCgacuugguag	1	1	JCK-3
uuggcgugagaNacuugguag	1	1	JCK-1
uuggcgugagagacuugguag	62	0	JCK-2
uuggcgugagagacuugguag	31	0	JCK-1
uuggcgugagagacuugguaA	2	1	JCK-1
uuggcgugagagacuugguagU	3	1	LT-1
uuggcgugagagacuugguagU	14	1	JCK-3
uuggcgugagagacuugguagC	1	1	JCK-1
uuggcgugagagacuugguagC	1	1	JT-2
uuggcgugagagacuugguagU	12	1	JCK-2
uuggcgugagagacuugguagU	18	1	JCK-1
uuggcgugagagacuugguagC	1	1	JCK-2
uuggcgugagagacuugguagC	5	1	JT-3
uuggcgugagagacuugguagU	12	1	LT-3
uggcgugagagGcuuggu	1	1	LT-3
uggcgugagagacuuggu	7	0	JCK-3
uggcgugagagacuugUu	1	1	JCK-2
uggcgugagagacuuggu	6	0	LT-3
uggcgugagagacuuggu	10	0	LCK-3
uggcgugagagacuuggu	2	0	LT-1
uggcgugagagacuuggu	6	0	JCK-2
uggcgugagagacuuggA	2	1	JCK-1
uggcgugagagacuuggu	6	0	LT-2
uggcgugagagacuuggu	8	0	JCK-1
uggcgugagagacuuggua	42	0	LCK-3
uggcgugagagacuugguC	4	1	LCK-3
uggcgugagagaAuuggua	1	1	LT-2
uggcgugagagacuuggua	50	0	JCK-2
uggcgugagagacuuggua	5	0	JT-3
uggcgugagagacuugguC	2	1	JCK-1
uggcgugagagacuuggua	41	0	LT-2
uggcgugagagacuugguU	2	1	JCK-1
uggcgugagagacuugguU	1	1	LT-3
uggcgugagagacuugguC	1	1	JCK-2
uggcgugagagacuuggua	47	0	JCK-3
uggcgugagagacuuggua	51	0	LT-3
uggcgugagagacuuggua	41	0	JCK-1
uggcgugagagacuugguU	6	1	JCK-2
uggcgugagagacuugguU	7	1	LCK-3
uggcgugagagacuuggua	12	0	JT-1
uggcgugagagacuuggua	30	0	LT-1
uggcgugagagacuuggua	9	0	JT-2
uggcgugagagacuugguag	29	0	JCK-3
uggcAugagagacuugguag	1	1	JCK-1
uggcgugagagacuugguag	20	0	LT-2
uggcgugagCgacuugguag	1	1	LT-3
uggcgugCgagacuugguag	1	1	JCK-3
uggcgugagagacuugguag	25	0	JT-1
uggcgugagagacuugguag	26	0	LT-3
uggcgugagagacuugguaA	3	1	LT-2
uggcgugagagacuugguaU	1	1	LT-1
uggcgugagagacuugguag	8	0	LCK-3
uggcgugagagacuugguag	13	0	LT-1
uggcgugagagacuugguag	23	0	JT-3
uggcgugagagacuugguag	35	0	JCK-1
uggcgugagagaAuugguag	1	1	LT-2
uggcgugagagacuugguag	38	0	JCK-2
uggcgugagagacuugguag	18	0	JT-2
uggcgugagagCcuugguaga	1	1	JCK-3
uggcgugagagacuugguagU	1	1	LT-3
uggcgugagagacuugguaga	42	0	LT-1
uggcgugagagacuugguaga	29	0	JT-1

Mature Star

agcgucacgugauaacgcguccuuugcuuuuuauuuuuuauuugaaauucaaaaaaauaauaauaaagauauauuuauauaauuguggaugugaaagaagaacuacuaccacuacagagagauuuuuuuaugaggcugagaaaauug**uuggcgugagagacuuggua***gaaaucuggguuuguuuggacacagaguuaggacuauucuuc****uuccgaguccgcccaugcc***aauaauuuccuccgucuuuuc			
uggcgugagagacuugguaga	14	0	JT-3
uggcgugagagacuugguaga	25	0	JCK-3
uggcgugagagacuugguaga	80	0	LT-3
uggcgugagagacuugguaga	6	0	JT-2
uggcgugagagacuugguaga	22	0	JCK-1
uggcgugagagacuugguaga	36	0	LCK-3
uggcgugagagacuugguaga	27	0	JCK-2
uggcgugCgagacuugguaga	1	1	LT-1
uggcgugagagacuugguagC	1	1	LT-3
uggcgugagagacuugguaga	54	0	LT-2
uggcgugagagacuugguagU	1	1	JCK-2
uggcgugagagacuugguagC	1	1	JCK-3
uggcgugagagacuugguagaa	2	0	JCK-1
uggcgugagagacuugguagaa	1	0	LT-1
uggcgugagagacuugguagaU	4	1	LT-3
uggcgugagagacuugguagaa	1	0	JCK-2
uggcgugagagacuugguagaU	4	1	LT-2
cgugagagacuugguaga	1	0	JCK-3
cgugagagacuugguagaaau	1	0	LT-2
cgugagagacuugguagaaauc	1	0	LT-3
cgugagagacuugguagaaaucu	1	0	LT-1
gugagagacuugguagaaauc	2	0	LT-2
ugagagacuugguagaaa	3	0	LT-3
ugagagacuugguagaaa	1	0	LT-1
ugagagacuugguagaaau	1	0	LT-3
ugagagacuugguagaaaucu	1	0	JCK-2
ugagagacuugguagaaaucuggg	2	0	JCK-2
ugagagacuugguagaaaucugggu	1	0	LT-1
acuugguagaaaucugggu	3	0	LT-3
acuugguagaaaucuggguuuguuug	3	0	JT-3
acuugguagaaaucuggguuuguuug	3	0	JT-2
cuugguagaaaucuggguuug	1	0	JCK-3
uggguuuguuuggacacagaguuag	1	0	LT-3
uuuguuuggacacagaguuaggacuau	1	0	JCK-2
uuuguuuggacacagaguuaggacuau	1	0	JCK-3
uuuggacacagaUuuaggacuauu	1	1	LT-3
uuggacacagaguuaggacu	1	0	JCK-3
uuggacacagaUuuaggacu	1	1	LT-1
uuggacacagaUuuaggacua	1	1	LT-1
uuggacacagaUuuaggacua	2	1	LT-2
uuggacacagaguuaggacuau	3	0	LCK-3
acacagaUuuaggacuauucuu	1	1	LT-3
cacagaUuuaggacuauu	2	1	LT-3
cacagaUuuaggacuauucu	2	1	LT-1
cacagaUuuaggacuauucuu	1	1	LT-3
cacagaUuuaggacuauucuu	1	1	JCK-1
cacagaUuuaggacuauucuu	2	1	JCK-3
cacagaUuuaggacuauucuuc	1	1	JCK-3
cacagaguuaggacuauucuucuucc	1	0	LT-3
acagaUuuaggacuauucuucu	2	1	JCK-1
acagaUuuaggacuauucuucu	1	1	JCK-3
cagaUuuaggacuauucuucu	1	1	JCK-3
cagaguuaggacuauucuucuuccgag	1	0	LT-1
uucuuccgaguccgcccaugcc	1	0	LT-3
ucuuccgaguccgcccaug	1	0	JCK-2
ucuuccgaguccgcccaug	3	0	LCK-3
cuuccgaguccgcccaugc	1	0	LT-3
cuuccgaguccgcccaugc	2	0	LT-2
cuuccgaguccgcccaugc	3	0	LCK-3
cuuccgaguccgcccaugU	2	1	LT-1
cuuccgaguccgcccaugc	1	0	LT-1
cuuccgaguccgcccaugcc	5	0	LCK-3
cuuccgaguccgcccaugcc	2	0	LT-1
cuuccgaguccgcccaugcc	3	0	LT-2
cuuccgaguccgcccaugcc	1	0	LT-3
cuuccgaguccgcccaugcca	1	0	LCK-3
cuuccgaguccgcccaugccaaua	3	0	JT-3
cuuccgaguccgcccaugccaaua	5	0	JT-2
uuccgaguccgcccaugc	88	0	LT-3
uuccgaguccgcccaugc	44	0	LT-2

Mature Star

```
agcgucacgugauaacgcguccuuugcuuuuuauuuuuuauuugaaauucaaaaaaauaauaauaaagauauauuuauauaauuguggaugugaaagaagaacuacuaccacuacagagagauuuuuuuaugaggcugagaaaauuguuggcgugagagacuugguagaaaucuggguuuguuuggacacagaguuaggacuauucuucuuccgaguccgcccaugccaauaauuuccuccgucuuuuc
.................................................................................................................................................................................................................uuccgaguccgcccaugc......................   2     0   JT-2
.................................................................................................................................................................................................................uuccgaguccgcccaugU......................   1     1   LT-1
.................................................................................................................................................................................................................uuccgaguccgcccaugc......................   5     0   JT-3
.................................................................................................................................................................................................................uuccgaguccgcccaugc......................   10    0   JCK-2
.................................................................................................................................................................................................................uuccgaguccgcccaugc......................   51    0   LT-1
.................................................................................................................................................................................................................uuccgaguccgcccaugc......................   12    0   JT-1
.................................................................................................................................................................................................................uuccgaguccgcccaugc......................   11    0   LCK-1
.................................................................................................................................................................................................................uuccgaguccgcccaugc......................   5     0   LCK-2
.................................................................................................................................................................................................................uuccgaguccgcccaugc......................   97    0   LCK-3
.................................................................................................................................................................................................................uuccgaguccgcccaugc......................   3     0   JCK-3
.................................................................................................................................................................................................................uuccgaguccgcccaugc......................   8     0   JCK-1
.................................................................................................................................................................................................................uuccgaguccgcccaugcc.....................   186   0   LT-3
.................................................................................................................................................................................................................uuccgaguccgcccaugcc.....................   36    0   JCK-3
.................................................................................................................................................................................................................uuccgaguccgcccaugcc.....................   41    0   JCK-2
.................................................................................................................................................................................................................uuccgaguccgcccaugcA.....................   1     1   LT-2
.................................................................................................................................................................................................................uuccgaguccgcccaugcc.....................   108   0   LT-2
.................................................................................................................................................................................................................uuccgaguccgcccaugcc.....................   29    0   JCK-1
.................................................................................................................................................................................................................uuccgCguccgcccaugcc.....................   1     1   LCK-3
.................................................................................................................................................................................................................uuccgaguccgcccaugcc.....................   14    0   JT-3
.................................................................................................................................................................................................................uuccgaguccgcccaugcc.....................   9     0   JT-1
.................................................................................................................................................................................................................uNccgaguccgcccaugcc.....................   1     1   JCK-1
.................................................................................................................................................................................................................uuccgaguccgcccaugcU.....................   8     1   LT-1
.................................................................................................................................................................................................................uuccgaguccgcccaugcU.....................   10    1   LCK-3
.................................................................................................................................................................................................................uuccgaguccgcccaugcU.....................   6     1   LT-3
.................................................................................................................................................................................................................uuccgaguccgcccaugcU.....................   1     1   JCK-2
.................................................................................................................................................................................................................uuccgaguccgcccaugcc.....................   160   0   LCK-3
.................................................................................................................................................................................................................uuccgaguccgcccaugcc.....................   10    0   JT-2
.................................................................................................................................................................................................................uuccgaguccgcccaugcc.....................   119   0   LT-1
.................................................................................................................................................................................................................uuccgaguccgcccaugcU.....................   2     1   JCK-1
.................................................................................................................................................................................................................uuccgaguccgcccaugcU.....................   16    1   LT-2
.................................................................................................................................................................................................................uuccgaguccgcccaugcca....................   59    0   LCK-3
.................................................................................................................................................................................................................uuccgaguccgcccaugccU....................   1     1   LT-2
.................................................................................................................................................................................................................uuccgaguccgcccaugcca....................   65    0   LT-3
.................................................................................................................................................................................................................uuccgaguccgcccaugcca....................   37    0   JT-1
.................................................................................................................................................................................................................uuccgaguccgcccaugcca....................   7     0   JT-3
.................................................................................................................................................................................................................uuccgaguccgcccaugcca....................   16    0   JCK-1
.................................................................................................................................................................................................................uuccgaguccgcccaugcca....................   28    0   JCK-2
.................................................................................................................................................................................................................uuccgaguccgcccaugccC....................   1     1   JCK-2
.................................................................................................................................................................................................................uuccgaguccgcccCugcca....................   1     1   LT-2
.................................................................................................................................................................................................................uuccgaguccgcccaugcca....................   40    0   LT-1
.................................................................................................................................................................................................................uuccgaguccgcccaugcca....................   33    0   LT-2
.................................................................................................................................................................................................................uuccgCguccgcccaugcca....................   1     1   JT-1
.................................................................................................................................................................................................................uuccgaguccgcccaugcca....................   19    0   JCK-3
.................................................................................................................................................................................................................uuccgaguccgcccaugcca....................   1     0   JT-2
.................................................................................................................................................................................................................uuccgaguccgcccaugccU....................   1     1   LT-3
.................................................................................................................................................................................................................uuccgaguccgcccaugccC....................   1     1   LT-2
.................................................................................................................................................................................................................uuccgaguccgcccaugcUa....................   1     1   LT-1
.................................................................................................................................................................................................................uuccgaguccgcccaugccaa...................   1     0   JCK-3
.................................................................................................................................................................................................................uuccgaguccgcccaugccaa...................   2     0   JCK-1
.................................................................................................................................................................................................................uuccgaguccgcccaugccaa...................   11    0   LT-1
.................................................................................................................................................................................................................uuccgaguccgcccaugccaa...................   12    0   LT-3
.................................................................................................................................................................................................................uuccgaguccgcccaugccaa...................   13    0   LT-2
.................................................................................................................................................................................................................uuccgaguccgcccaugccaU...................   1     1   LT-1
.................................................................................................................................................................................................................uuccgaguccgcccaugccaa...................   24    0   LCK-3
.................................................................................................................................................................................................................uuccgaguccgcccaugccaa...................   16    0   LCK-1
.................................................................................................................................................................................................................uuccgaguccgcccaugccaa...................   3     0   JCK-2
.................................................................................................................................................................................................................uuccgaguccgcccaugccaa...................   19    0   LCK-2
.................................................................................................................................................................................................................uuccgaguccgcccaugccaau..................   115   0   JCK-2
.................................................................................................................................................................................................................uuccgaguccgcccaugccaau..................   91    0   JCK-3
.................................................................................................................................................................................................................uuccgCguccgcccaugccaau..................   1     1   JCK-3
.................................................................................................................................................................................................................uuccgaguccgcccaugccUau..................   1     1   JCK-1
.................................................................................................................................................................................................................uuccgaguccgcccaugccaau..................   19    0   LT-2
.................................................................................................................................................................................................................uuccgaguccgcccaugccaau..................   62    0   LT-3
.................................................................................................................................................................................................................uuccgaguccgcccaugccaCu..................   1     1   JCK-2
.................................................................................................................................................................................................................uuccgaguccgcccaugccUau..................   2     1   JCK-2
.................................................................................................................................................................................................................uuccgaguccgcccaugccaau..................   48    0   LT-1
.................................................................................................................................................................................................................uuccgaguccgcccaugcAaau..................   1     1   JCK-3
.................................................................................................................................................................................................................uucUgaguccgcccaugccaau..................   1     1   JCK-3
.................................................................................................................................................................................................................uuccgaguccgcccaugccaau..................   10    0   LCK-3
.................................................................................................................................................................................................................uuccgaguccgcccaugccaau..................   26    0   LCK-1
```

```
                                                                                                                                                                          Mature                                                         Star
agcgucacgugauaacgcguccuuugcuuuuuauuuuuuauuugaaauucaaaaaaauaauaauaaagauauauuuauauaauuguggaugugaaagaagaacuacuaccacuacagagagauuuuuuuaugaggcugagaaaauuguuggcgugagagacuugguagaaaucuggguuuguuuggacacagaguuaggacuauucuucuuccgaguccgcccaugccaauaauuuccuccgucuuuuuc
.................................................................................................................................................................................................................uuccgaguccgcccaugccaau...................   76   0   JCK-1
.................................................................................................................................................................................................................uuccgaguccgcccaugccaau...................   14   0   LCK-2
.................................................................................................................................................................................................................uuccgCguccgcccaugccaau...................   2    1   JCK-2
.................................................................................................................................................................................................................uuccgaguccgcccaugccaauU..................   4    1   LCK-3
.................................................................................................................................................................................................................uuccgaguccgcccaugccaauU..................   27   1   JCK-3
.................................................................................................................................................................................................................uuccgaguccgcccaugccaauU..................   16   1   JCK-1
.................................................................................................................................................................................................................uuccgaguccgcccaugccaauU..................   24   1   JCK-2
.................................................................................................................................................................................................................uuccgaguccgcccaugccaaua..................   1    0   LT-2
.................................................................................................................................................................................................................uuccgaguccgcccaugccaauU..................   23   1   LT-1
.................................................................................................................................................................................................................uuccgaguccgcccaugccaauU..................   8    1   LT-2
.................................................................................................................................................................................................................uuccgaguccgcccaugccaauU..................   17   1   LT-3
.................................................................................................................................................................................................................uuccgaguccgcccaugccaauUa.................   2    1   JCK-1
.................................................................................................................................................................................................................uuccgaguccgcccaugccaauUa.................   1    1   LT-3
.................................................................................................................................................................................................................uuccgaguccgcccaugccaauUa.................   3    1   JCK-3
.................................................................................................................................................................................................................uuccgaguccgcccaugccaauUa.................   4    1   JCK-2
.................................................................................................................................................................................................................uuccgaguccgcccaugccaauUau................   1    1   LT-1
.................................................................................................................................................................................................................uuccgaguccgcccaugccaauaUu................   1    1   LT-1
..................................................................................................................................................................................................................uccgaguccgcccaugcc......................   1    0   LT-1
..................................................................................................................................................................................................................uccgaguccgcccaugcc......................   1    0   JCK-3
..................................................................................................................................................................................................................uccgaguccgcccaugcca.....................   1    0   LT-1
..................................................................................................................................................................................................................uccgaguccgcccaugcca.....................   1    0   JCK-3
..................................................................................................................................................................................................................uccgaguccgcccaugccaa....................   2    0   LT-2
..................................................................................................................................................................................................................uccgaguccgcccaugccaau...................   1    0   LT-2
..................................................................................................................................................................................................................uccgaguccgcccaugccaau...................   2    0   JCK-3
..................................................................................................................................................................................................................uccgaguccgcccaugccaau...................   2    0   JCK-2
..................................................................................................................................................................................................................uccgaguccgcccaugccaaua..................   5    0   JCK-2
..................................................................................................................................................................................................................uccgaguccgcccaugccaaua..................   2    0   JCK-3
..................................................................................................................................................................................................................uccgaguccgcccaugccaauU..................   1    1   JCK-2
..................................................................................................................................................................................................................uccgaguccgcccaugccaaua..................   4    0   JCK-1
...................................................................................................................................................................................................................ccgaguccgcccaugccaaua..................   2    0   JCK-3
...................................................................................................................................................................................................................ccgaguccgcccaugccaaua..................   1    0   JCK-2
..................................................................................................................................................................................................................................ccaauaauuuccuccgucu.....   1    0   LT-3
```
